# A coordinated multidisciplinary model of care is needed for child and family centered care in pediatric genetic cancer risk services: a scoping review

**DOI:** 10.1007/s10689-025-00474-8

**Published:** 2025-06-20

**Authors:** Andrew M. Grant, Natalie Taylor, Jane Maguire, Sharon de Graves, Christina Signorelli, Noemi A. Fuentes-Bolanos, Katherine M. Tucker, Marilyn Cruickshank

**Affiliations:** 1 Sydney Children’s Hospital, Sydney, Australia; 2 University of Technology Sydney, Sydney, Australia; 3 UNSW Sydney, Sydney, Australia; 4 Maridulu Budyari Gumal (SPHERE), Sydney, Australia; 5 University of Technology Sydney, Sydney, Australia; 6 VCCC Alliance, Melbourne, Australia; 7 University of Melbourne, Melbourne, Australia; 8 UNSW Sydney, Sydney, Australia

**Keywords:** Cancer predisposition, Advanced practice nursing, Pediatrics, Child and family centred care

## Abstract

Cancer remains a leading cause of death in children/adolescents. Approximately 8–18% of children/adolescents with cancer have an underlying pediatric Genetic Cancer Risk (p-GCR). P-GCR clinics offer surveillance aimed at improving survival outcomes. Yet children/adolescents require more than surveillance protocols to support holistic health. A multidisciplinary model of care (MoC), including Advanced Practice Nurses (APN) is needed. Yet a MoC and formal description of the APN is lacking in p-GCR clinics. To explore existing evidence of holistic, multidisciplinary approaches to care for children/adolescents and families with a p-GCR; to identify how Advanced Practice Nurses (APN) contribute to care delivery in p-GCR services. A scoping review was conducted in three databases: MEDLINE (Ovid), Embase (Ovid) and CINAHL Complete. JBI methodology for conducting and reporting scoping reviews was used to search MEDLINE, Embase and CINAHL Complete. Gray and white literature was considered from 1991 to 2023. Thirty two studies met inclusion criteria. Thirteen aspects of a MoC in p-GCR were identified including: clinic scope, clinic locality, clinicians involved, care coordination, clinic activity, geography, centralisation of care, psychosocial aspects, shared decision making, education, referrals, transition to adult services and research. There were 10 APN roles described that supported the service/organisation and the delivery of holistic care to children/adolescents with a p-GCR. Using a systematic approach, this review identified how services provide care to children/adolescents with a p-GCR and the APN role in these services. A multidisciplinary MoC with dedicated care coordination can enable child and family centred care with a holistic healthcare approach.

**Supplementary Information:**

The online version contains supplementary material available at 10.1007/s10689-025-00474-8.

## Background

Cancer remained one of the leading causes of death in children/adolescents worldwide [1]. Precision medicine programs in pediatric cancer are providing an increasing array of targeted treatment options that can improve the survival rates of children/adolescents with cancer. However, this remains a reactive medical approach, with cancers often found at a late stage and once they have metastasized. Identifying those most at risk of cancer can help identify cancers as early as possible, or prevent lesions becoming malignant in some circumstances [2, 3]. There is a growing recognition that germline genetics influences some children/adolescents’ risk of cancer. Pediatric Genetic Cancer Risk (p-GCR), also termed pediatric Cancer Predisposition Syndromes, refer to conditions that predispose children/adolescents to solid tumours and hematological malignancies due to a germline pathogenic/likely pathogenic variant [4, 5]. 

It is currently estimated that between 8 and 18% of children/adolescents with cancer have a p-GCR [3, 6–8]. Additionally, children/adolescents without cancer may be diagnosed with a p-GCR following genetic testing due to a known GCR in the family or other physical or neurocognitive features suggestive of an underlying genetic cause [9]. Although individually rare, the number of children/adolescents diagnosed with a p-GCR is becoming increasingly recognized. This is influenced by the increasing use of genomic sequencing, rapidly advancing technology, and expansion of genomic studies in many pediatric centres internationally [10, 11]. 

Following a p-GCR diagnosis children/adolescents are likely to be offered surveillance aimed at improving survival outcomes [3]. This is achieved by early cancer identification, through screening with physical assessments, medical imaging and/or pathology tests at regular intervals to monitor for signs and symptoms of cancer. Surveillance protocols have demonstrated improved overall survival rates for certain p-GCR [12, 13]. However cancer surveillance can be burdensome physically, socially, and psychologically (Box 0). Additionally, p-GCR may be associated with non-cancer health implications such as neurodivergence, that might be highly impactful during childhood [14]. For reasons above, it is clear the holistic value-based health of a child/adolescent with a p-GCR is not encapsulated in the context of cancer screening alone. A comprehensive approach to managing their care requires more than imaging to support overall health and wellbeing.

Due to the rarity, complexity and diversity of these genetic conditions, a multidisciplinary approach with clinicians familiar with specific p-GCRs is important. This includes dedicated patient and family-centred follow-up clinics with internationally recognized surveillance protocols, genetic counselling, patient and family education, cascade genetic testing, individualized risk assessment and management, and health promotion. Involving Advanced Practice Nurses (APN) could enable a coordinate approach with child/adolescent and family centred care.

**Box 1:** ..

Examples of Surveillance Burden in children with a p-GCR [3, 15–18]
Physical • Blood tests can be traumatic. • MRIs and other imaging may require a general anaesthetic in young children. • False positive results can lead to additional interventions (e.g. biopsy)
Social • Frequency of screening events (some p-GCR require 3–4 monthly screening) • Time away from family and friends • Time away from school or work • Travel distance to attend centralized expert centres. • Financial pressures
Psychological • “Scanxiety”– screening may serve as a reminder of cancer risk. • Repeated imaging, assessments or pathology can be traumatic for young children. • Social isolation (see social) can impact mental health. • Incidental findings can lead to additional psychological stress

APNs extend their scope as registered nurses through formal education and extensive practice to provide specialist care with expert knowledge and skills in a dedicated area of practice. APNs demonstrate leadership capabilities with complex decision making skills across healthcare settings with both a patient and organisation focus [19, 20]. Although APNs have facilitated comprehensive healthcare coordination of children/adolescents with chronic and complex conditions, a formal description of APNs in p-GCR is lacking.

Additionally, a multidisciplinary Model of Care (MoC) that describes how services can holistically meet the needs of children/adolescents and families with a p-GCR has not been published. Initial literature searching found limited references describing how services deliver care to children/adolescents and families with a p-GCR. Studies were heterogenous in nature, with variations in areas such as the type of p-GCR seen, what the service offered and how the service was run. The studies were of low level of evidence, primarily including case reports and review articles. There was no literature comprehensively describing a MoC for children/adolescents with a p-GCR. A preliminary search of the Cochrane Database of Systematic Reviews and Open Science Framework was conducted in February 2023 and no current or in-progress scoping reviews or systematic reviews on MoC, service delivery, or the APN role related to p-GCR were identified.

This scoping review explored p-GCR service delivery in relation to different aspects of a MoC. In doing so, the review aims to build the foundations for a holistic approach to care for children/adolescents and families with a p-GCR. Inclusive in the MoC is an outline of the role of an Advanced Practice Nurse (APN) in p-GCR.

### Review questions

1. What aspects of models of care for children/adolescents with a p-GCR have been described in the literature?
2. How do advance practice nurses contribute to the delivery of care in p-GCR clinics?

## Methods

The scoping review was conducted in accordance with JBI scoping review methodology [21]. Reporting of the scoping review followed the Preferred Reporting Items for Systematic Reviews and Meta-Analysis Protocol extension for Scoping Reviews (PRISMA-ScR) [22]. The scoping review protocol was first registered in Open Science Framework (OSF) osf.io/axkp7/ and was published in JBI Evidence Synthesis [23]. 

### Inclusion/exclusion criteria

#### Population

The scoping review included studies involving children/adolescents, up to the age of 21 years, with a GCR. Studies reporting only on adults, or where the age group is not identified, were excluded. Data reporting on children/adolescents as part of a mixed adult and pediatric population was included if the pediatric data was clearly distinguishable.

APNs were considered for inclusion as a separate population. APNs perform an extension of standard nursing skills in dedicated positions gained through additional education and experience specific to their field of practice [24]. APN positions considered include nurse practitioners, nurse consultants, nurse specialists, nurse coordinators and nurse navigators. Non-APN roles were excluded.

#### Concept

This scoping review included articles that described:

1. aspects of a MoC for healthcare delivery. This included information that directly or indirectly impacts on how the health service functioned and/or provided care.
2. the role of an APN. This included clinical and non-clinical responsibilities as well as patient facing and health service roles.

#### Context

This review considered healthcare services that provided clinical care to individuals with a GCR. Both dedicated GCR clinics and programs that included GCR streams were considered.

#### Types of sources

All study designs were considered including quantitative, qualitative, and mixed methods studies from both grey and white literature. All forms of review, such as systematic, umbrella and scoping reviews were included. Additionally, opinion papers, letters to the editor, conference papers, dissertations, and theses were considered for inclusion.

Studies published in languages other than English were excluded due to limitations of resourcing for translation services. The review limited the search to studies published after January 1991. In 1991, the link between the APC gene and familial adenomatous polyposis (FAP), the first described p-GCR, was published [25]. 

#### Deviations from the protocol

The original protocol considered barriers and facilitators of care for children/adolescents with a p-GCR and gaps in care delivery as two additional review questions. The search strategy, study selection, data extraction, and data analysis were conducted as described in the a priori protocol with deviation regarding the grey literature search described below [23]. Following data analysis, it was decided that describing barriers, facilitators and gaps did not align with the objectives of this review and detracted from the key questions being explored. Principally, these did not provide further information or insights into a broad understanding of care delivery for children/adolescents with a p-GCR internationally. Additionally, “barriers, facilitators and gaps” were not part of the search terms described in the a priori protocol. Therefore, a deviation from the protocol was needed to focus on the two review questions included in this scoping review.

A further deviation from the protocol was made, with the exclusion of a dedicated grey literature and website search as sources for this scoping review. Following consideration of the level of evidence available, including grey literature and website review was felt to further detract from the scientific rigour of the scoping review.

### Search strategy

The electronic database search followed a three-step process. Electronic databases included MEDLINE (Ovid), Embase (Ovid) and CINAHL Complete (EBSCOhost). An initial search was undertaken in MEDLINE to identify articles on the topic and build a greater understanding of the concepts being explored. Text words contained in the article titles and abstracts, and key words were used to develop a full search strategy in MEDLINE. This followed an iterative process to optimize specificity and sensitivity of the search. The search used keywords, index terms and Medical Subjects Headings (MeSH). A research librarian was consulted to help develop the search terms and translate search terms between databases. The complete search strategies are provided in Appendix I. The reference lists of included articles were examined for relevant secondary sources. References of all included articles were analysed to identify any additional literature relevant to the review.

### Study selection

**Fig. 1:**
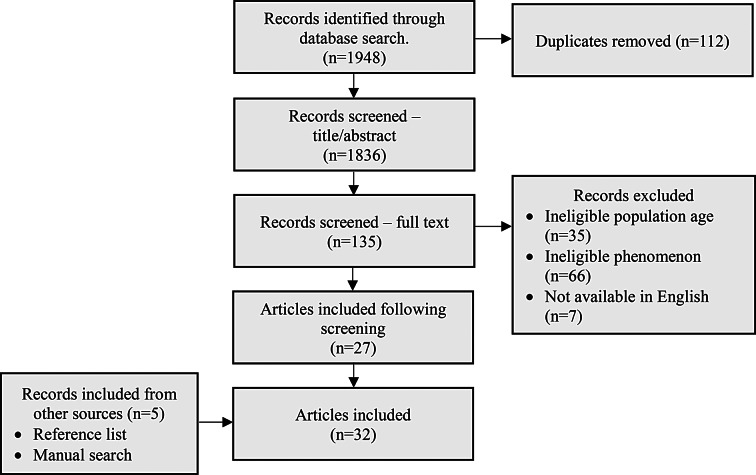
PRISMA diagram

Following the search, all identified citations were uploaded into EndNote V.20 (Clarivate Analytics, PA, USA) and duplicates removed. Remaining citations were uploaded into JBI System for the Unified Management, Assessment and Review of Information (JBI SUMARI; JBI, Adelaide, Australia).^29^ Following pilot testing of 50 citations, two researchers (AG and CS) independently screened the title and abstract of the remaining citations against inclusion criteria. The full text of the potentially relevant articles were retrieved and screened against inclusion criteria independently by the same researchers. The reasons for exclusion were recorded and summarized (Figure 1). All disagreements that arose between reviewers were resolved through discussion.

### Data extraction

Data were extracted from included articles using a data extraction tool as recommended by JBI methodology for scoping reviews [21]. The extraction table was developed by authors and presented in the original protocol [23]. The data extraction table included citation details and specific information about the population, concepts and context, study methodology and relevant findings related to the scoping review. The data extraction tool was initially piloted by two authors (AG & CS) independently with results compared and discussed. This was initially performed with the first included article, then four additional articles at which point consistency between authors was found. Following piloting, the first author (AG) completed data extraction of the remaining articles. Finally, two authors (AG & CS) extracted data on a sixth article chosen at random, to ensure consistency remained.

### Data analysis and presentation

**Fig. 2:**
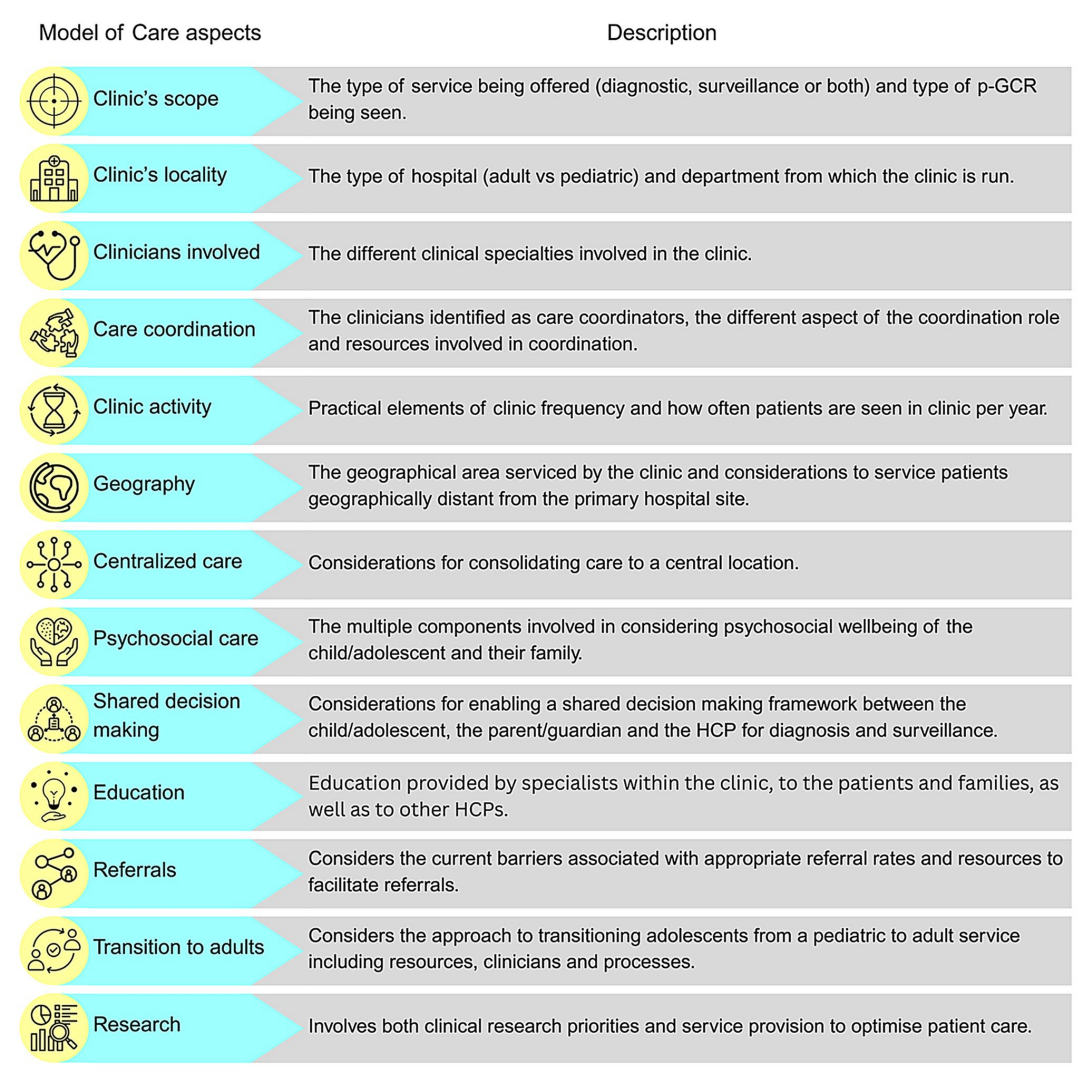
Pediatric Genetic Cancer Risk Clinic: Model of Care. p-GCR = pediatric genetic cancer risk, HCP = healthcare professionals

The study selection results are illustrated using a PRISMA flow diagram (Fig. 1). The findings are presented in tabular and graphical formats to align with the scoping review questions. Aspects of the MoC are visually represented (Fig. 2). A narrative summary accompanies the findings and describes how the results relate to the review’s objective and questions.

## Results

### Database search

A total of 1948 records were retrieved through the database search. Once duplicates were removed, two reviewers (AG and CS) screened titles and abstracts then full text articles against the inclusion criteria. A total of 27 articles were included from the database search. One author (AG) undertook a bibliographic search of included articles and performed a manual search. Five additional articles met inclusion criteria. Figure 1 presents a PRISMA flow diagram of the search strategy and inclusion process.

Two articles report commentary on the same organisation. Authors of the scoping review agreed to include both articles as they provide different perspectives and report separate discussions. However, to prevent duplication, descriptions of the aspects of the MoC were extracted from Shea et al. [26] and the APN involvement was extracted from Hemenway et al. [27]

### Characteristics of included articles

**Fig. 3:**
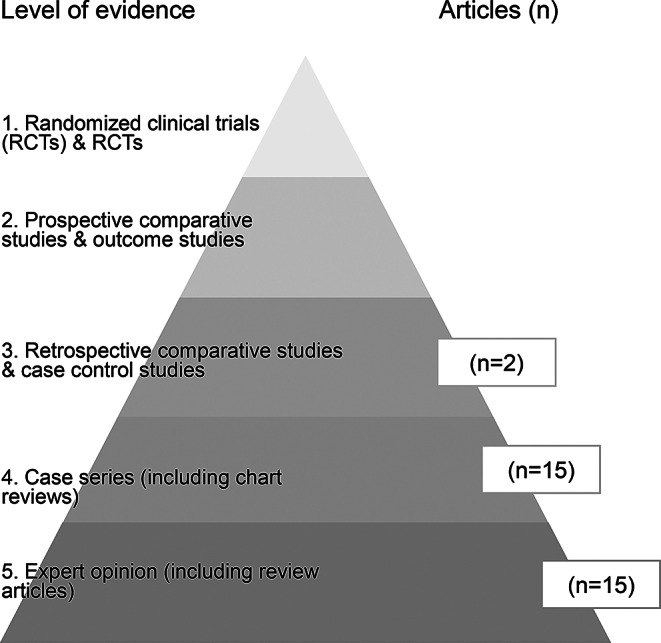
Level of evidence of included articled based on Bittner 2014

Articles were published between 1991 and 2024, with the majority (*n* = 26/32, 81%) published from 2017 onwards. Studies took place in the USA (*n* = 16), Australia (*n* = 3), Canada (*n* = 3), the UK (*n* = 2), Spain (*n* = 2) and one each in Brazil, Germany, Greece, Ireland, Italy and Portugal. Overall, articles were of a low level of evidence as demonstrated in Fig. 3. The study designs included chart reviews (*n* = 9), literature reviews (*n* = 8), discussion papers (*n* = 7), cross-sectional research (*n* = 3), multimethod research (*n* = 3), a cohort study (*n* = 1), and qualitative interviews (*n* = 1).

### Review findings

The findings are presented in two sections to address the two scoping review questions.

#### Research question 1: what aspects of models of care for children/adolescents with a p-GCR have been described in the literature?

Articles provided information that have the potential to inform key elements required to provide/facilitate optimal care delivery and models of care for this population. Further, pending the geographical location and scope of service, there were some limited descriptions of specific models of care. One example is the ‘Hub and Spoke’ model described by Llyod et al. [28] where a centralized NF1 specialty centre (the Hub) facilitated care locally and aided the provision of care through smaller centres (the Spokes) dispersed throughout the United Kingdom. Another example is the transition model described by Peron et al. [29] for adolescents moving to adult TSC services. These two examples highlight the diversity between included articles, yet al.so represent the only two articles clearly explained the model used to provide care. As such, MoC aspects have been extracted and consolidated to build an understanding of what is known in the literature (Table 1).

**Table 1:** Summary of findings

First author (year)	Aims/purpose	Study design	Model of Care aspects
Al-Sarhani (2022)	To discuss the role of imaging in cancer predisposition syndrome screening.	Literature review	*Centralisation of care* with specialty clinic *Psychosocial aspects*: Recognise financial pressures and emotional burden *Shared decision making* with local imaging *Research*: Clinical research priorities
Alsowat (2020)	To determine if children referred to the TSC Clinic at the Hospital for Sick Children were receiving appropriate surveillance before being seen in the clinic.	Chart review	*Clinic’s scope*: Follow-up and surveillance *Clinic’s locality*: Children’s hospital *Psychosocial aspects*: Recognise financial pressures *Transition to adult services*: Planned for dedicated transition pathway *Research*: Clinical research priorities
Attard (2008)	To describe the authors experience with children with FAP who are younger than 10 years at the time of presentation.	Cross-sectional research	*Clinic’s scope*: Diagnostic service and follow-up and surveillance *Clinic’s locality*: Adult’s hospital *Care coordination* by APN; Use of data registry *Transition to adult services*: Partnership between paediatric and adult service *APN role*: Care coordination
Barnett (2021)	To synthesize and characterize the existing LFS literature on psychosocial outcomes, educational needs, support services, and available interventions for patients and families across the lifespan, from childhood through adulthood.	Literature review	*Psychosocial aspects*: Recognise emotional burden; Psychological evaluation and interventions through referral to external clinicians; Patient peer support *Shared decision making* including child and caregiver; use of developmentally appropriate communication *Education*: Child and family directed education *Transition to adult services*: GC re-engage prior to transition
Black (2021)	(1) To describe demographic characteristics of the retrospective study and predisposition clinic study; (2) To assess the changes in the screening tool between the retrospective study and predisposition clinic study; (3) To assess possible differences between the retrospective study and the predisposition clinic study	Chart review	*Clinic’s scope*: Follow-up and surveillance *Clinic’s locality*: Children’s hospital; Haematology/oncology department *Referrals*: Use referral guidelines; Recommended formal referral process
Davies (2018)	To describe the importance of screening programs for children with inherited endocrine neoplasia syndromes. To identify the role of a paediatric endocrine nurse specialist.	Discussion paper	*Clinic’s scope*: Follow-up and surveillance *Care coordination* by APN; Use of data registry *Transition to adult services*: Partnership between paediatric and adult service; APN facilitated transition *APN role*: Care coordination; Consultant; Educator; Researcher; Collaborator; Leader; Change Agent; Patient advocate; Liaison; Clinical expert
Druker (2017)	To present recommendations for addressing issues specific to pediatric cancer genetics.	Literature review	*Psychosocial aspects*: Recognise emotional burden; Psychological evaluation and interventions through referral to external clinicians *Shared decision-making* including child and caregiver; Surveillance tailored to child and family needs; Use of developmentally appropriate communication *Education*: Child and family directed education *Transition to adult services*: GC re-engage prior to transition; GC facilitated transition
Escudero (2022)	To present the clinical experience of a multidisciplinary team focused on identifying families with CPS to improve their diagnostic rate, clinical management, and genetic counselling.	Chart review	*Clinic’s scope*: Diagnostic service *Clinic’s locality*: Adult’s hospital *Referrals*: Use referral guidelines
Grossen 2022)	(1) To assess different multidisciplinary clinic layouts utilized in centers worldwide; (2) To characterize an institutional experience with the management of these conditions, focusing on the patient demographics, clinical presentation, complications, and therapeutic strategies seen in a patient population.	Multi-methods research (Literature review and chart review)	*Clinic’s scope*: Follow-up and surveillance *Clinic’s locality*: Children’s hospital *Care coordination*: Use of data registry *Clinic activity*: Bi-monthly clinic; Multiple specialists see patients in tandem or consecutively. *Geography*: Clinic services entire state (USA) *Centralisation of care* with specialty clinic *Shared decision making* in care delivery *Transition to adult services*: Dedicated transition pathway; Partnership between paediatric and adult service *Research*: Clinical research priorities
Groves (2019)	To characterize the features of a CPS clinic population and quantify the need for specific therapeutic interventions.	Multi-methods research (Cross sectional research and chart review)	*Clinic’s scope*: Diagnostic service and follow-up and surveillance *Clinic’s locality*: Children’s hospital; Haematology/oncology department *Care coordination*: Use of data registry *Research*: Clinical research priorities
Hemenway (2014)	*To describe the role of a nurse coordinator in a multidisciplinary neuroendocrine clinic*	Discussion paper (abstract)	*Care coordination* by APN (Refer to Shea et al. For additional aspects of the MoC) *APN role*: Care coordination; Education; Collaborator; Communication; Managing resources
Huber (2021)	To determine the number and clinical characteristics of children and adolescents affected by CPS in a tertiary-care children’s hospital; (2) To compare current hospital practice with the surveillance recommendations and, if necessary, adjust practice to recommendations.	Cohort study	*Clinic’s scope*: Follow-up and surveillance *Clinic’s locality*: Children’s hospital; Haematology/oncology department *Education*: Child and family directed education; HCP directed education
Hynds (2017)	To develop a service ensuring children had access to a multidisciplinary clinic on an annual basis and to create a registry of patients which captures the incidence and prevalence of NF1 in Ireland.	Discussion paper (abstract)	*Clinic’s scope*: Follow-up and surveillance *Clinic’s locality*: Children’s hospital; General paediatrics department *Care coordination* by APN; Use of data registry *Clinic activity*: Monthly clinic; Annual patient review *Education*: HCP directed education *APN role*: Care coordination; phone service
Kokkinou (2019)	To present the 3 year experience of a multidisciplinary neurocutaneous disorder clinic with a focus on the most frequent type of NCS, NF1.	Chart review	*Clinic’s scope*: Follow-up and surveillance *Clinic’s locality*: Children’s hospital; General paediatrics department *Clinic activity*: Monthly clinic *Geography*: Clinic services central Greece and many Greek islands *Referrals*: Use referral guidelines *Transition to adult services*: Planned for dedicated transition pathway
Leedman (2021)	To describe the experience of a surveillance clinic for children with, or at risk of hereditary cancer predisposition syndromes	Discussion paper	*Clinic’s scope*: Diagnostic service and follow-up and surveillance *Clinic’s locality*: Children’s hospital; General paediatrics department *Clinic activity*: Quarterly clinic; Six monthly patient review
Levine (2010)	To examine parental attitudes and beliefs regarding endoscopic surveillance and genetic testing in minors at risk for developing FAP.	Multi-methods research (Cross sectional research and qualitative research)	*Clinic’s scope*: Diagnostic service and follow-up and surveillance *Clinic’s locality*: Adult’s hospital; Haematology/oncology department
Lin (2020)	*To describe the multidisciplinary testing and management of basal cell nevus syndrome.*	Literature review	*Clinic’s scope*: Follow-up and surveillance *Care coordination* by case worker *Education*: Child and family directed education *Research*: Clinical research priorities
Lloyd (2018)	(1) To present the current structure within which care of NF2 patients delivered in England; (2) To discusses what benefits and challenges have developed as a result of centralisation.	Literature review	*Clinic’s scope*: Follow-up and surveillance *Clinic’s locality*: Hospitals network *Care coordination* (clinician not specified); Use of data registry *Clinic activity*: Weekly clinics; Annual patient review *Geography*: National network (UK) *Centralisation of care* using ‘Hub and spoke’ model *APN role*: Patient advocate; Communication
MacDonald (2000)	To describe the application of genetic testing of children for hereditary cancers and the resultant ethical and psychosocial implications.	Literature review	*Clinic’s scope*: Diagnostic service *Psychosocial aspects*: Recognise emotional burden; Psychological evaluation and interventions by clinicians in the clinic; Evaluation of the families’ beliefs and values; Patient peer support *Shared decision making* including child and caregiver; use of developmentally appropriate communication *Education*: Child and family directed education *APN role*: Education; Patient Advocate; Communication; Clinical Expert; Psychosocial support; Managing resources
McGill (2019)	To evaluate families’ psychosocial experiences following pediatric cancer-related genetic counselling/testing, their satisfaction with genetic services, and their information and support needs.	Qualitative interviews	*Clinic’s locality*: (1) Children’s hospital; Haematology/oncology department (2) Adult’s hospital; Hereditary medicine department *Psychosocial aspects*: Recognise emotional burden; Psychological evaluation and interventions through referral to external clinicians *Shared decision making* including child and caregiver; Consider child led approach; Use of developmentally appropriate communication *Education*: Child and family directed education
Merker (2018)	(1) To describe the current availability and utilization of specialty NF services in the U.S; (2) assess for any potential disparities in access based on patient’s age, disease type, or region of residence.	Chart review	*Clinic’s scope*: Follow-up and surveillance *Clinic’s locality*: Hospitals network *Education*: HCP directed education *Transition to adult services*: Partnership between paediatric and adult service
Mitchell (2019)	To review two of the emerging childhood cancer predisposition syndromes, DICER1 syndrome and rhabdoid tumour predisposition syndrome, including the clinical phenotype and published surveillance recommendations, as well as what is known about the psychosocial implications of the diagnosis of a CPS in a child.	Literature review	*Education*: Child and family directed education *Transition to adult services*: GC re-engage prior to transition
North (1993)	To describe the clinical characteristics of the first 200 patients seen in the NF1 clinic.	Chart review	*Clinic’s scope*: Follow-up and surveillance *Clinic’s locality*: Children’s hospital; Neurology department *Clinic activity*: Twice weekly clinic; Annual patient review *Geography*: Clinic services entire state (Australia) *Centralisation of care* with specialty clinic *Psychosocial aspects*: Recognise emotional burden; Psychological evaluation and interventions by clinicians in the clinic
Ordóñez (2021)	To evaluate the outcome of prophylactic thyroidectomies (PT) in patients with MEN 2 syndrome in a tertiary centre.	Chart review	*Clinic’s scope*: Diagnostic service *Clinic’s locality*: Adult’s hospital *Centralisation of care* with specialty clinic
Parsons (2021)	*To describe the role of the Pediatric Nurse Practitioner in relation to Li Fraumeni Syndrome*	Discussion paper	*Care coordination* by APN *Psychosocial aspects*: Recognise emotional burden; Psychological evaluation and interventions by clinicians in the clinic *Education*: Child and family directed education; HCP directed education *APN role*: Care coordination; Education; Patient advocate; Communication; Clinical expert; Psychosocial support
Peron (2018)	To review existing models of transition in different conditions, discuss our experience, and propose general rules to follow when establishing a transition program for TSC.	Literature review	*Clinic’s locality*: Adult’s hospital; Neurology department *Care coordination* by physician *Clinic activity*: Once-twice weekly clinic *Education*: HCP directed education *Transition to adult services*: Dedicated transition pathway; Partnership between paediatric and adult service: Physician facilitated transition
Rebelo (2023)	(1) to describe the organization of the MOCND and evaluate the first 5 years. (2) to share our institutional experience and (3) to analyze the advantages of a multidisciplinary center and approach in NCS.	Chart review	*Clinic’s scope*: Diagnostic service and follow-up and surveillance *Clinic’s locality*: Children’s hospital *Care coordination* by physician *Clinic activity*: Weekly clinic *Geography*: Clinic services southern Portugal *Psychosocial aspects*: Recognise emotional burden; Psychological evaluation and interventions by clinicians in the clinic; Patient peer support *Education*: Child and family directed education; HCP directed education *Transition to adult services*: Partnership between paediatric and adult service
Samuel (2014)	To present authors’ perspectives on how the scope of practice in the management of families of children with cancer has changed in the era of genomic analysis as a result of next-generation sequencing techniques and cancer-surveillance strategies.	Discussion paper	*Psychosocial aspects*: Recognise financial pressures
Sandy (2020)	To describe the experience of a Brazilian tertiary center in the diagnosis and follow-up of pediatric patients with PJS	Chart review	*Clinic’s scope*: Diagnostic service and follow-up and surveillance *Psychosocial aspects*: Recognise financial pressures
Shea (2016)	*To describe the experience of a Neurofibromatosis Clinic*	Discussion paper (abstract)	*Clinic’s scope*: Diagnostic service and follow-up and surveillance *Clinic’s locality*: Children’s hospital; Neurology department *Care coordination* *Clinic activity*: Monthly clinic *Education*: Child and family directed education
Venier (2022)	To investigate current referral practices of US-based HCP involved in the care of pediatric oncology patients with a suspected CPS with a focus on referral guideline usage.	Cross-sectional research	*Referrals*: Use referral guidelines
Wiener (2017)	To describe the overall usability of the Distress Thermometer and symptom checklist with youth (ages 7– 21) living with NF1.	Cross-sectional research	*Psychosocial aspects*: Recognise emotional burden

Thirteen aspects were extracted, each of which is described below. The aspects were: the clinic’s scope, clinic’s locality, clinicians involved, care coordination, clinic activity, geography, centralisation of care, psychosocial aspects, shared decision making, education, referrals, transition to adult services and research. These aspects fall into two broad care components: Child and Family Centred Care (CFCC) and health service provision.

### Clinic’s scope (CFCC & health service provision)

The clinic’s scope was described by 21 articles in relation to the overall service provided. This was described in three key ways. First, three articles described a diagnostic service, offering genetic testing, risk evaluation, and risk reduction or surveillance recommendations, however, did not include patient follow-up [16, 30, 31]. Second, 11 articles described the clinic providing on-going medical assessments, follow-up and surveillance for children/adolescents already diagnosed with a p-GCR [5, 28, 32–40]. Third, seven articles included both the diagnostic service and the on-going medical follow-up in their clinic’s scope [26, 41–46]. 

There were differences in the types of p-GCR seen in the clinic (Fig. 2). Ten clinics focused on a specific p-GCR [26, 28, 29, 31, 32, 36, 40, 41, 43, 46], five clinics included neuroendocrine genetic cancer risks [34, 35, 37, 39, 45], while eight clinics included all types of GCR [5, 16, 18, 30, 33, 42, 47]. Huber et al. [5] documented 41 different types of GCR seen in their service, which included Trisomy 21 and neurofibromatosis type 1 (NF1). However, Groves et al. [42] excluded Trisomy 21 and NF as these populations were served by other clinics within the organisation.

### Clinic’s locality (health service provision)

Departments providing the p-GCR clinic included; haematology/oncology (*n* = 6) [5, 18, 30, 33, 42, 44], general pediatrics (*n* = 3) [36, 37, 43], neurology (*n* = 3) [26, 29, 40] and hereditary medicine (*n* = 2) [18, 41]. Huber et al. [5]. explored where patients received care in a cohort of 272 individuals with a p-GCR. These patients presented to 20 different departments and 29% received care from multiple departments.

The majority of clinics were held in children hospitals (*n* = 13) [5, 18, 26, 32, 33, 35–37, 40, 42, 43, 45, 47], with six clinics held in adult hospitals [18, 29–31, 41, 44]. There were two articles describing a hospitals network which included both adult and children hospitals [28, 39]. 

### Clinicians involved (health service provision)

**Fig. 4:**
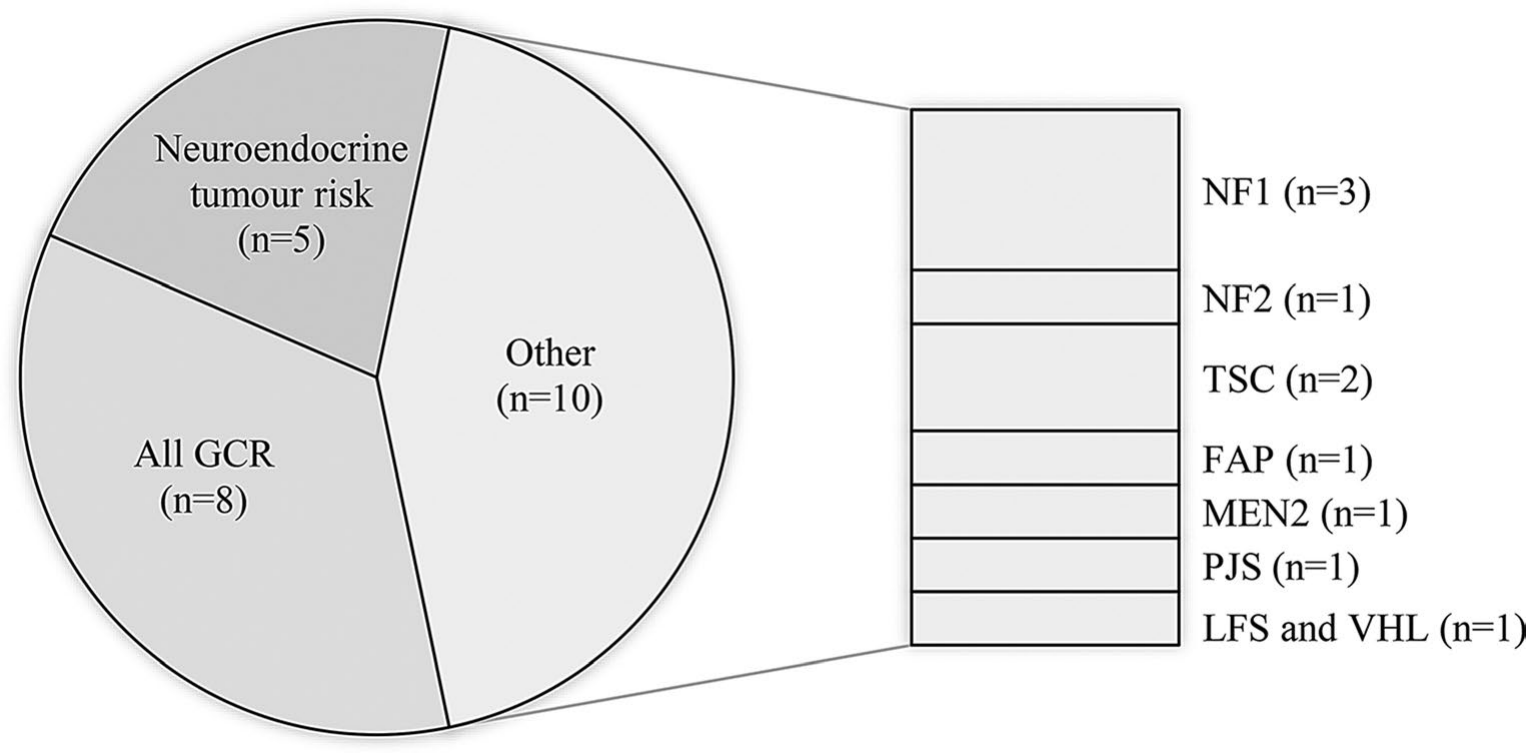
p-GCR types described in clinics. NF1 = neurofibromatosis Type 1; NF2 = neurofibromatosis Type 2; TSC = Tubular Sclerosis Syndrome; FAP = Familial Adenomatous Polyposis; MEN2 = Multiple Endocrine Neoplasia type 2, PJS-Peutz-Jeghers Syndrome; LFS = Li Fraumeni Syndrome; VHL = von Hippel Lindau disease

Multiple clinicians were involved in the provision of care across clinics. The number of clinicians involved ranged from two [48] to fifteen [29]. The lead physician differed depending on the department and the type of p-GCR managed within the clinic. For example, a neurologist was described as the lead physician in a clinic seeing children/adolescents with Tuberous Sclerosis Complex through an epilepsy centre [29]. Alternatively, an oncologist was the lead physician in a clinic seeing all types of p-GCR through a pediatric oncology department [30]. Fig. 4 shows all HCPs listed in the clinics. Three studies described the benefit of patients seeing multiple care providers during a single clinic visit. This was performed either in tandem or consecutively [35, 45, 49]. The studies suggested that this led to a reduction in cancellation and ‘no-show’ rates due to patient convenience, including saving time and money, and was particularly beneficial for rural patients who have to travel long distances for these services. Groves et al. [42] reviewed the provision of allied health for children/adolescents with a p-GCR, identifying high use of physiotherapy, occupational therapy and speech therapy.

### Care coordination (CFCC & health service provision)

Care coordinators were described as important for the effective functioning of clinics. Coordinator disciplines were reported in seven clinics and varied including APNs [27, 34, 36, 41, 48], physicians [29, 45], case workers [38] or not specified [28]. Care coordinators facilitated communication between clinical teams through various methods including maintaining patient documentation, organized interdisciplinary team meetings and through direct collaboration with different clinicians for a shared patient approach [27, 38, 45]. Care coordinators prepared clinical information prior to each appointment by reviewing medical notes, collating previous results and making them available for relevant clinicians [27, 28]. Care coordinators also promoted patient compliance through regular follow up to aid with surveillance including imaging and clinic appointments [27–29, 45]. Hemenway et al. [27] described additional responsibilities of the nurse coordinator in establishing an NF1 multidisciplinary program. These were not listed in regular care provision in other articles and will be described further in the section on APN contribution.

The use of a data registry was described in six articles [28, 34–36, 41, 42]. This facilitated care delivery by having a structured way to track when surveillance events were due with alerts and reminders for the clinician coordinating care. The registry was also described in the context of prospective data collection to facilitate research, collaborations, and ongoing review of clinical processes for continual improvement.

### Clinic activity aspects (health service provision)

Eight articles described the frequency of the clinic, ranging from twice weekly to four times per year [26, 28, 29, 35–37, 40, 43, 45]. Articles describing clinics for neurocutaneous conditions [28, 36, 40] stated patients were reviewed at least yearly. Leedman et al. [43] stated patients were seen every six months in their clinic which primarily saw patients with LFS.

### Geographical aspects (health service provision)

The expansive geographical area covered by clinics was described by five studies. Two clinics serviced an entire state in the USA [35] or Australia [40]. Kokkinou et al. [37] described a clinic servicing Central Greece including Athens and many Greek islands. The outpatients’ clinic for neurocutaneous conditions described by Rebelo et al. [45] covered the southern portion of Portugal. Lloyd et al. [28] described a clinical network, with four tertiary ‘hub’ hospitals, supported by additional smaller ‘spoke’ hospitals, delivering care nationally to patients with NF2. Spoke hospitals provided clinical care for patients living near them in conjunction with the hub hospital. Hub hospitals provided clinical support to the spoke hospitals and were responsible for supervising patient care.

### Centralized care aspect (health service provision)

Four studies describe the value of centralizing care of children/adolescents and adolescents with a p-GCR [28, 31, 40, 47]. This was described as important because concentrating the management of patients to specialty clinics allows clinicians the opportunity to build expertize in monitoring and treating patients with a GCR. Al-Sarhani et al. [47]. recommended interpretation of whole body MRIs to be performed by a centralized collection of radiologists as lesions in children/adolescents with a p-GCR need familiarity with the condition.

Lloyd et al. [28] claimed that the centralized care network improved patient outcomes in care delivery and quality of life for individuals with NF2. Lloyd et al. [28] and Grossen et al. [35] also found their centralized networks acquired centralized funding. The funding structure provided greater access to certain interventions in treatment and management as well as greater advocacy for lower-income patients.

### Psychosocial aspects (CFCC)

The psychosocial implications of a p-GCR diagnosis were described in 12 articles [15, 16, 18, 40, 45, 48–50]. McGill et al. [18] identified feelings of social and emotional isolation from caregivers, with limited connection to others who have the same p-GCR. Feelings of parental guilt for passing on the genetic condition and the impacts on family relationships were also voiced. Druker et al. [15] described the anxiety of having scans and other surveillance events given the reminder of cancer risk around this time.

Adolescence was recognized as an key stage requiring psychosocial support [18]. A flexible approach to care during adolescence that continued to meet their changing needs was recommended. This included greater information requirements, independence and autonomy.

Seven articles advocate for psychological evaluation and interventions to support the child and family through the clinic [15, 16, 18, 40, 45, 48, 49]. This was either with psychological support embedded in the clinic (*n* = 4) [16, 40, 45, 48] or through referrals to psychology specialists external to the clinic (*n* = 3) [15, 18, 49]. Barnett et al. [49] described a process of addressing psychological concerns by genetic specialists, with a process to refer if required.

North [40] and MacDonald and Lessick [16] found that open and realistic discussions with families about health risks in the context of the child helped to dispel misconceptions that could otherwise fuel anxiety. MacDonald and Lessick [16] also advocated for an evaluation of the families’ beliefs and values to help address psychosocial needs. Additionally, it was recommended to have a planned approach for potentially emotionally distressing conversations such as the return of genetic results. For example, discussing results with caregivers initially to allow for their emotional reaction. This was followed by discussing results with the child with caregivers present.

In addition to the supports offered by healthcare professionals, three articles suggested there was benefit in connecting with others who have the same genetic condition. This may be through patient support groups or less formally with families who have been seen in the same clinic [16, 45, 49]. MacDonald and Lessick [16] described this support in relation to both a national support group for caregivers, as well as establishing links between teenagers with the same diagnosis. Furthermore, the results indicated that these relationships could help address feelings of isolation.

Financial pressures families face when attending clinics and utilising treatment options were described in four articles [32, 46, 47, 51]. Families also described the inconvenience and negative impact of time spent away from work and school to attend clinic appointments [51]. No practical processes were described in the article to address these pressures.

### Shared decision making (CFCC)

Shared decision making was documented as an important part of care delivery [15, 16, 18, 35, 47, 49]. Four articles encouraged a shared decision making approach, which included both the child and caregivers [15, 16, 18, 49]. Further to this, McGill et al. [18] found that some caregivers advocated for a child led approach to decision making for adolescents when considering genetic testing. In addition to shared decision making in genetic testing, a collaborative approach to surveillance was seen as valuable. Druker et al. [15] suggested that surveillance, including imaging and frequency, should be tailored to meet the needs of the child and family. Al-Sarhani et al. [47] considered enabling imaging at local facilities to reduce the families’ burden of coming to hospital.

The collaborative approach was supported through relationships between HCP and family members built on a foundation of trust [15, 18, 35]. Additionally, to enable shared decision making, children/adolescents and their caregivers need to be adequately informed. The use of developmentally appropriate language and strategies were recommended to facilitate counselling methods, information sharing, discussion of concerns and space to ask questions [15, 16, 18, 49]. 

### Education aspects (CFCC and health service provision)

The provision of education was described in relation to both patient and family education [5, 15, 16, 18, 26, 38, 45, 48, 49, 52] and HCP education [5, 29, 36, 39, 45, 48]. Child and family education required the use of developmentally appropriate language that is adapted to the needs of the family [15, 16, 18, 49]. Barnett et al. [49] suggested this could be facilitated by HCPs delivering small pieces of information over multiple meetings to promote greater understanding. Misconceptions and understanding about genetic concepts proved challenging for some HCPs to advocate for genetic testing and follow-up [16, 44, 48, 51]. This was seen as an important aspect when addressing the patients’ healthcare needs.

Articles described a process of educating clinicians within the team through discussion of complex cases, review of current literature or self-directed learning [29, 45, 48]. Robelo et al. [45] described the multidisciplinary approach facilitated discussions for a more comprehensive understanding of the disease and increased their capacity to collaborate with international experts on difficult cases. Hynds et al. [36] found that having clinicians attend an established service to learn processes through observation of the clinic helped build knowledge when establishing a new NF1 clinic. More broadly, Huber et al. [5]. advocate for education and awareness to be widely published and distributed to HCPs regarding cancer risk and surveillance for children/adolescents with a p-GCR. Merker et al. [39] discussed the value of collegial connections between clinics and community groups more generally, identifying benefits such as greater awareness, improved access and increased referrals to the clinic.

### Referrals to a p-GCR clinic (health service provision)

Referral rates to a pediatric cancer genetics clinic were seen as suboptimal [30, 31, 33, 53]. Black [33] estimated that up to 40% of patients with a potential p-GCR were not referred and Ordonez et al. [31] found that many children/adolescents were referred at an age older than recommended. In a cross-sectional analysis of pediatric oncologists, Venier et al. [53] found that HCPs did not refer children/adolescents diagnosed with cancer for genetic assessment for multiple reasons including priority and lack of available genetics services. Black [33] suggests that patients are not referred because risk factors are not being identified.

Screening tools, including referral guidelines, were commonly used to help clinicians identify patients who may be eligible for risk assessment and genetic testing [30, 33, 37, 53]. Referrals were made by multiple providers both within the clinic’s health service and externally, including parents’ associations, specific p-GCR associations or pediatricians [37, 40, 45]. Shea et al. [26] suggested that as their clinic grew, the frequency of appropriate referrals increased.

No article describes a formal referral process, although Black [33] recommended that this was important to achieve appropriate referral rates.

### Transition to adult services (CFCC and health service provision)

Transition to adult services was identified as a key aspect of continuous patient care. This was described as a structured and planned process over time, which required transition specific resourcing. Two studies described their clinic as having a dedicated pathway for the transition of adolescents to an adult service [29, 35]. Two additional studies [32, 37] describe this transition pathway as a future plan for their clinic.

Six studies stated the importance of partnering between pediatric and adult services [29, 34, 35, 39, 41, 45]. Peron et al. [29] described a model with a close professional relationship between pediatric and adult clinicians. Both clinical teams had access to the pediatric and adult medical records and specialists met regularly to discuss transitioning patients. Patients were seen in both the pediatric and adult service during transition, which was a strategy also used by Rebelo et al. [45]

Starting the transition process at an early age was seen as important to build knowledge, empower the transitioning patient and establish relationships between the receiving adult service [18, 29, 45]. 

Barnett et al. [49] and McGill et al. [18] described supportive relationships built through ongoing care can facilitate a smooth transition to adult services. A transition coordinator was seen as important to facilitate the organisation of uninterrupted care between pediatric and adult services. However the discipline of who took on the transition coordinator varied, with either a physician [29], specialist nurse [34] or genetic counsellor [15] performing this function. Regardless of the coordinator position, genetic counsellors were seen as an integral part of this process through re-engaging adolescents and families prior to transition [15, 49, 52]. 

### Research (health service provision)

Three studies stated that future research into service delivery and/or patient related outcomes was needed [35, 38, 42]. Future directions in service delivery and evaluation included research into time to treatment, number of interventions per patient, patient referrals, and how to optimize the clinics’ evaluation. Patient related research outcomes included clinical trials into treatment options, further exploration of patient reported outcomes and overall life expectancy and survival rates for individuals with a GCR [32, 35, 42]. One study described the need to standardize imaging protocols to ensure consistency in evaluating and reporting on MRIs for children/adolescents with a GCR [47]. 

#### Research question 2: how do advance practice nurses contribute to the delivery of care in p-GCR clinics?

**Fig. 6:**
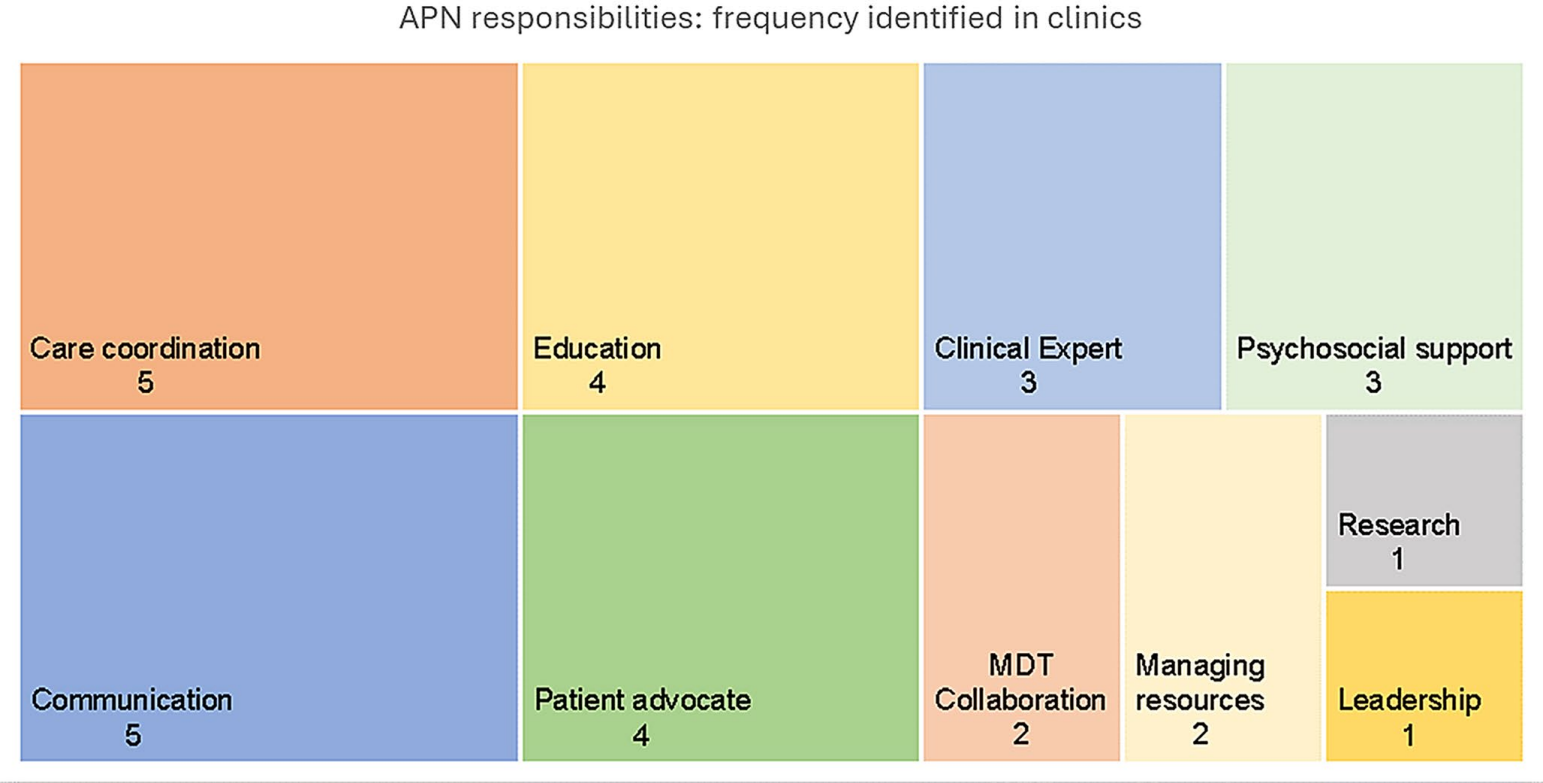
Advanced Practice Nurse (APN) responsibilities identified

Seven articles identified a nurse as part of the p-GCR clinic in an advanced practice or coordination role [16, 27, 28, 34, 36, 41, 48]. Of these, five articles described aspects of how the APN contributes to care delivery [16, 27, 28, 34, 48]. There were 10 specific responsibilities listed in the articles (Fig. 6). However, only four of these were described in detail including coordination, communication, education and psychosocial support.

### Nurse coordinator

Five articles identified coordination as part of the APN role [27, 34, 36, 41, 48]. In addition to the coordinator description above, Hemenway et al. [27] described coordination in relation to establishing the service within the hospital. In collaboration with multidisciplinary teams, the APN identified the clinic’s goals, considered the infrastructure required and developed practical resources to facilitate smooth running of the clinic.

### Communication

Five studies describe communication with patients and carers is an important component APNs provide for ongoing care [16, 27, 28, 34]. Llyod and Evans [28] assert that nurses are a valuable point of contact for patients and carers. Furthermore, they state that the nursing role has improved communication between patients and clinicians with two-way sharing of relevant information. Two studies include communication and liaison with the multidisciplinary team into the APN role [16, 34]. 

### Education

Patient education is identified as part of the APN role by four studies [16, 27, 34, 48]. The education components identified include informing both the child/adolescent and caregivers on benefits and risks of genetic testing, disease processes, clinical care, medication management and provision of patient information literature. MacDonald and Lessick [16] suggest that education forms part of the holistic care APNs provide children/adolescents and their families.

### Psychosocial support

Three studies identify psychosocial support as a valuable APN role [16, 34, 48]. MacDonald and Lessick [16] describe APNs as bringing a ‘*human touch*’ to healthcare, particularly with discussions surrounding genetic testing and the integration of technology in medicine. The studies acknowledged nurses can assist in facilitating the delivery of information in a cognitively and developmentally targeted way to support children/adolescents and carers. APNs were also described as enabling ongoing discussions by building psychological safety into care delivery [16, 34]. Interactions with community groups as a way of engaging with and supporting patients was also identified as part of the APN role in pediatric cancer genetics [16, 34]. 

## Discussion

This review incorporated information on p-GCR services internationally. There were 13 aspects identified around how services deliver care to children/adolescents and families with a p-GCR. These aspects could be considered integral to the design of a multidisciplinary MoC. Additionally, ten responsibilities that make up the APN role were identified, which complement care delivery in p-GCR clinics. This adds to our understanding of a child and family centred approach to value-based care specific for this population and highlights the importance of integrating APNs within the MoC.

**Fig. 5:**
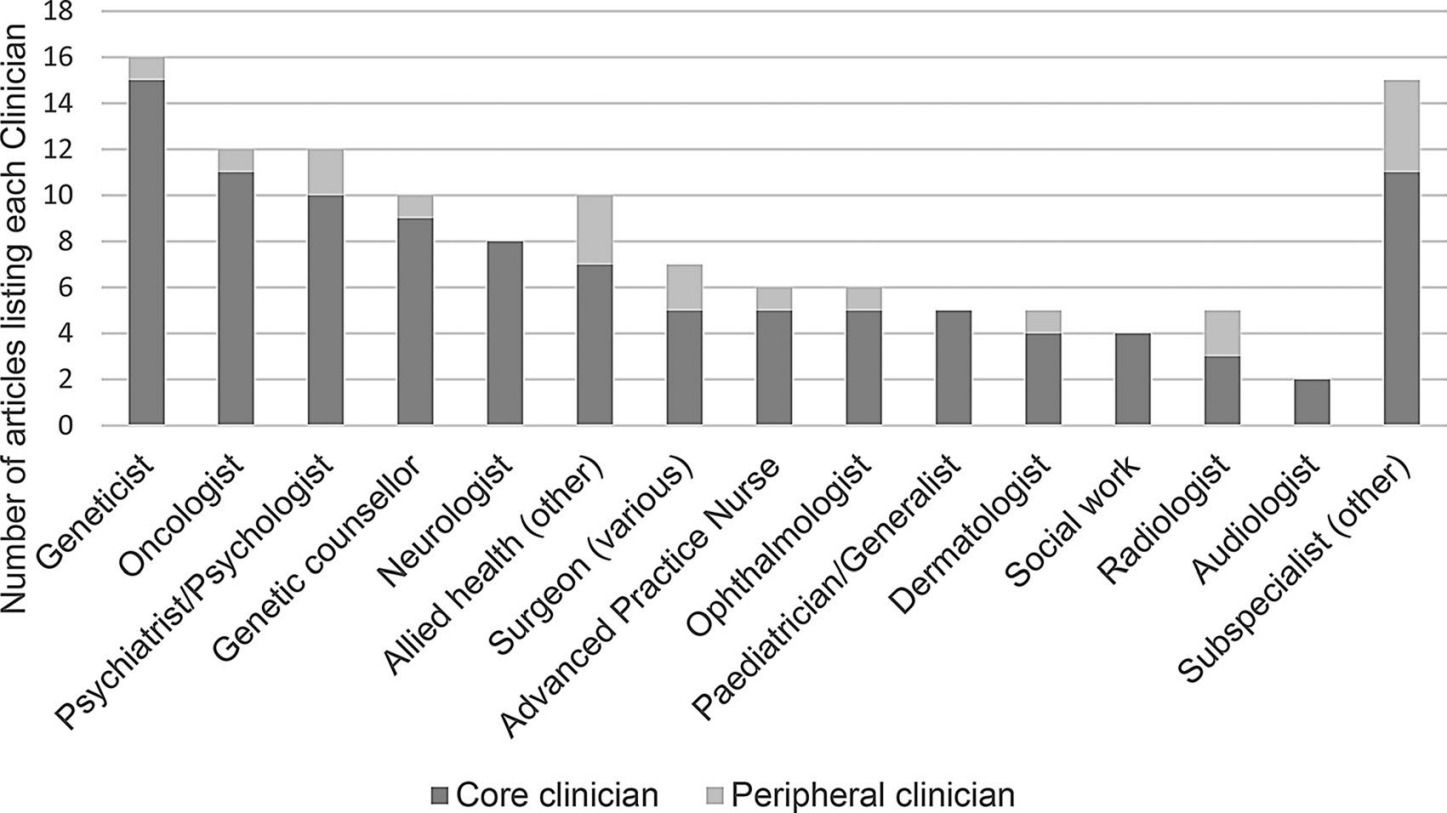
Clinicians involved in pediatric Genetic Cancer Risk clinics

Children/adolescents with a p-GCR and their caregivers require coordinated care for their rare, complex, and chronic illness. Yet these families experience challenges in accessing care that comprehensively meets their needs [5, 40, 44, 49]. As a step towards addressing some of these challenges, this scoping review identified and consolidated the various aspects of a p-GCR MoC. Delineating aspects of a MoC can enable services to deliver a holistic approach that addresses the needs of the child/adolescent and family, and is equitable and evidence based [54, 55]. Two primary components of a p-GCR service were demonstrated in the literature including; CFCC and health service provision (Fig. 5).

Core Clinicians are those directly involved in cases. Peripheral Clinicians are interdisciplinary teams who are involved in care when required.

### Child and family centred care

CFCC has been described as both a MoC and a philosophy in pediatrics, which advocates for a partnership between the child/adolescent, their family and HCPs. It recognizes essential family support structures while acknowledging the child/adolescent’s unique perspective and rights to informed decision making about their healthcare [56]. APNs have long been recognized for facilitating CFCC in many healthcare settings [20, 57–60]. In the context of p-GCR, our review highlighted the value APNs can add, particularly through care coordination, providing psychosocial support and delivering education.

The scoping review identified the importance of a care coordinator within the clinic, with the responsibility primarily undertaken by an APN. Children/adolescents with chronic rare diseases, including those with p-GCR, require longitudinal management of health and wellbeing, often involving multiple clinicians and teams across different health services. Navigating and negotiating these health requirements can be challenging, particularly when access pathways are unclear or absent. Lacking integrated care that spans different health services can lead to fragmented care with poor adherence to clinical recommendations [61–63]. As such, coordination is a central aspect of a p-GCR MoC.

In unison with coordinating clinical needs, it is imperative that the child/adolescent and family’s psychosocial needs are addressed. The literature identified potential stressors such as scan related anxiety, the burden of surveillance and the risk or event of tumours and/or malignancy [15, 16, 18]. APNs were found to incorporate psychological safety into clinical discussions and facilitate links with both professional and community groups who may support the psychosocial wellbeing of the children/adolescents and families. However, there were few articles in the literature that incorporated dedicated clinicians involved in psychosocial care. This gap is an important consideration for CFCC in p-GCR services.

Facilitating informed decision making by empowering children/adolescents and families through education can help alleviate some anxiety [15, 16, 49, 61, 63]. As with other rare diseases, there is a scarcity of information on p-GCR that is credible, relevant and current [62]. Additionally, primary HCPs can face challenges with the continual progress of knowledge on these diverse conditions and caregivers may encounter HCPs unfamiliar with the p-GCR diagnosis [64]. Therefore, it is important a p-GCR service can facilitate targeted education to children/adolescents and families. APNs have the skills and resources to engage in person centred education, which is a core competency with their scope of practice [20, 57, 65]. The review found APNs provided education in p-GCR clinics [16, 27, 34, 48], with the method of regular small pieces of information [49] aligning with how APNs engage with patients.

### Heath service provision

Internal service structures are key to the function and service delivery by p-GCR services. These structures can identify how care is delivered by a service efficiently and effectively. It is advantageous for the internal service structures to be flexible to ensure they meet the needs of both the patient population and consider the specific healthcare context. APNs are well positioned to enable the establishment and regular review of healthcare services. The skills and unique scope within healthcare, positions APNs to provide leadership and guidance with this, as described by Hemenway et al. [27]

The range of clinicians involved in the delivery of care was also seen as an essential consideration to service provision. In addition to genetic clinicians who were described in all clinics, the literature suggests that the type of p-GCR influences the core clinicians involved, due to the clinical care requirements. For example, neurologists and developmental pediatricians are important in neurofibromatosis clinics. These patients present with neurodevelopmental comorbidities, which are often the primary clinical concern during childhood [26, 35, 36, 39]. Conversely, LFS has a significant risk of malignancy with no associated neurodevelopmental comorbidities. Therefore, oncologists are important clinicians in the care of these patients [43, 48, 49]. Additionally, the clinical diversity and complexity of p-GCR often requires the input of multiple allied health clinicians [28, 35, 38, 42]. The review found that clinics tended to involve an MDT with clinicians who have expertize with p-GCR.

For clinicians to develop expertize in p-GCR, it is important to combine experience with training. Due to the rarity of p-GCR, the literature indicated that gaining experience in this field is often achieved through a centralized MoC, as with other rare diseases [66, 67]. Additionally, the literature identifies that a centralized service allows for case discussions and facilitates international collaborations to leverage expert knowledge [28, 31, 47]. 

The review showed that primary healthcare providers and subspecialists also play an invaluable part in caring for children/adolescents with a p-CGR [64]. An holistic MoC must integrate collaborations with these external providers as well as informing and supporting the care they deliver. Identifying appropriate means to offer expertize broadly and generally, as well in a targeted approach, should be a key consideration for p-GCR services.

As well as empowering primary physicians to deliver optimal care, building p-GCR knowledge more generally can support appropriate referral rates. The review identified that clinical services could benefit from further enhancing referral processes. Inappropriate referral rates can lead to delayed diagnosis and suboptimal clinical management. Diagnostic delay is a common challenge faced by families with a rare genetic disease [64, 68]. In the case of p-GCR, this can lead to delayed cancer diagnosis in the child/adolescent, which may limit treatment options. Additionally, there are wider implications for the entire family who may also harbour the same genetic change. Establishing referral processes is therefore essential for p-GCR services. Yet further research is needed to identify how this can be effectively achieved, as despite current attempts, referrals to many p-GCR services remain a barrier to care delivery [31, 33, 53]. 

The transition from pediatric to adult services is an important, yet challenging time for individuals with a p-GCR. It is commonly described as a priority across health services, yet young people continue to be lost to follow-up during transition [29, 69]. APNs are shown to be valuable throughout the transition process, providing education, advocacy, supporting collaboration and communication and guiding structured processes. The review identified the importance of a *planned* and *purposeful* transition [29, 35, 37]. The transition should be a collaboration between the pediatric and adult services, involving the young person and relevant support people [29, 70]. Essentially, the goal is to provide uninterrupted healthcare that empowers the young person to autonomously manage their healthcare needs.

### The APN in p-GCR

As described above, the APNs roles can be linked to key functions and responsibilities that support a holistic p-GCR MoC. Considering that APNs have shown to be essential in multiple clinical settings for many years, the dearth of literature describing APNs in p-GCR services is striking. Yet this is not uncommon in the field of genetics more broadly. In a cross-sectional study of nurse leaders across 18 countries, Calzone et al. identified that only five countries involved a specialized genetics nurse role [71]. This may be the consequence of limited genetic education in both undergraduate and post graduate nursing curricula as well as educational resources clinically. Building on these findings, Tonkin et al. presented a Roadmap to facilitate the integration of genomics into nursing practice [72]. Three key elements to achieve this were identified through the research including; resources, leadership and collaboration. Empowering nurse leaders to act as clinical experts and establish international collaborations can help to address this gap. Considering APNs in p-GCR, this network would be considerably smaller than general genetics. However, there remains the potential to leverage larger genomics nursing networks to help establish formalized roles more generally, with dedicated knowledge requirements specific to each setting [65, 71, 72]. 

### Limitations

There was heterogeneity in the type of articles included, the clinical condition reviewed, and service provided. The review did not include nursing articles if their position was not identified as a form of advanced practice. This may have excluded relevant articles. A systematic quality appraisal of the articles was not performed, and the level of evidence included was low according to the GRADE model. Only articles written in English were included which would exclude research on p-GCR from non-English speaking parts of the world. Furthermore, there was a higher representation of clinical practices from the USA and underrepresentation from low resource settings. As such, the scoping review does not make recommendations for clinical practice.

## Conclusion

The purpose of this scoping review was to explore what aspects of a MoC for children/adolescents with a p-GCR have been described in the literature. The review found commonalities established to facilitate care delivery that focused on both the health service itself, and the children/adolescents and families to which care is provided. The review had a particular focus on the APN role in p-GCR clinics. Although there is a striking lack of literature, the review found that APNs contribute meaningfully to p-GCR clinics, supporting the MoC through service development and service delivery using a CFCC approach.

It is important for p-GCR clinics to reflect on service delivery and the MoC they have established, as well as considering areas for future development. The results of this scoping review may be used as a framework to reflect on different aspects of a MoC relevant to the healthcare context and the clinic’s patient population to ensure the provision of value-based healthcare. However, further research exploring the patient experience and patient outcomes is needed to clearly delineate a MoC that comprehensively provides CFCC effectively and equitably to children/adolescents with a p-GCR and their families.

### Implications for research

Variation between services remains in what care is offered, how it is delivered and who provides the care. The level of evidence identified in this review is markedly low and there is a dearth of literature describing the APN role. The review offers foundations of a multidisciplinary p-GCR MoC involving an APN, broadly identifying key aspects relevant to a service. Research to systematically explore children/adolescents and family’s needs in this context will help identify areas for value-based service delivery. Additionally, evaluating the effectiveness of programs is needed to establish evidence for the outcomes of p-GCR MoC.

## Electronic supplementary material

Below is the link to the electronic supplementary material.

Supplementary Material 1

## Data Availability

No datasets were generated or analysed during the current study.
