# Supplementary material for: A coordinated multidisciplinary model of care is needed for child and family centered care in pediatric genetic cancer risk services: a scoping review
Source: Fam Cancer. 2025 Jun 20;24(3):55. doi: 10.1007/s10689-025-00474-8 (PMC12181217; doi:10.1007/s10689-025-00474-8)
Supplement: Supplementary file 1 — Supplementary Material 1 [file 10689_2025_474_MOESM1_ESM.docx]

| **MEDLINE Search strategy (search updated 2 June, 2023)** | | |
| --- | --- | --- |
| 1 | exp adolescent/ or exp child/ or exp infant/ or (infant disease* or childhood disease*).ti,ab,kf. or (adolescen* or babies or baby or boy? or boyfriend or boyhood or child* or girl? or infant* or juvenil* or kid? or minors or minors* or neonat* or neonat* or newborn* or new-born* or paediatric* or peadiatric* or pediatric* or perinat* or preschool* or puber* or pubescen* or school* or teen* or toddler? or underage? or under-age? or youth*).ti,ab,kf,hw. | Population #1 |
| 2 | exp Genetic Predisposition to Disease/ or  Germ-Line Mutation/ or  germline.tw/ or  exp Neoplastic Syndromes, Hereditary/ or  familial cancer.tw/ or  (cancer adj2 predisposition).tw/ or | Concept #1 |
| 3 | (nurse or nurses or nursing).tw/ or  nursing, team/ or  exp Nurses/ or  exp Nursing/ or  exp Advanced Practice Nursing/ or  exp Nurse Clinicians/ or | Concept #2 |
| 4 | care coordination.tw/ or  surveilla*.tw/ or  exp "Delivery of Health Care, Integrated"/ or  disease management/ or  multidisciplinary.tw/ or  exp Patient Care Management/ or  exp Patient Care Planning/ or  ((model or models) adj2 care).tw/ or  "continuity of patient care"/ or  transition to adult care/ | Concept #3 |
| 5 | health services/ or adolescent health services/ or community health services/ or health services for persons with disabilities/ or nursing services/ or patient care/ or personal health services/ or preventive health services/ or  exp genetic services/ or  clinic.tw/ or  exp hospital/ or  (treatment adj2 (centre or center)).tw | Context |
| 6 | 1 and 2 and (3 or 4) and 5 |  |
| 7 | limit 6 to yr="1991 - 2023" |  |

| **EMBASE Search strategy (search updated 2 June, 2023)** | | |
| --- | --- | --- |
| 1 | exp adolescent/ or exp child/ or exp infant/ or (infant disease* or childhood disease*).ti,ab,kf. or (adolescen* or babies or baby or boy? or boyfriend or boyhood or child* or girl? or infant* or juvenil* or kid? or minors or minors* or neonat* or neonat* or newborn* or new-born* or paediatric* or peadiatric* or pediatric* or perinat* or preschool* or puber* or pubescen* or school* or teen* or toddler? or underage? or under-age? or youth*).ti,ab,kf,hw. | Population #1 |
| 2 | exp Genetic Predisposition/ or  Germline Mutation/ or  germline.tw/ or  exp Herditary tumor syndrome/ or  familial cancer.tw/ or  (cancer adj2 predisposition).tw/ or | Concept #1 |
| 3 | (nurse or nurses or nursing).tw/ or  nursing, team/ or  exp Nurse/ or  exp Nursing/ or  exp Advanced Practice Nurse/ or  exp clinical nurse specialist/ or | Concept #2 |
| 4 | care coordination.tw/ or  surveilla*.tw/ or  exp integrated healthcare system/ or  disease management/ or  multidisciplinary.tw/ or  exp Patient Care/ or  exp Patient Care Planning/ or  ((model or models) adj2 care).tw/ or  transition to adult care/ | Concept #3 |
| 5 | health services/ or  exp genetic service/ or  clinic.tw/ or  exp hospital/ or  (treatment adj2 (centre or center)).tw | Context |
| 6 | 1 and 2 and (3 or 4) and 5 |  |
| 7 | limit 6 to yr="1991 - 2023" |  |

| **CINAHL Search strategy (search updated 2 June, 2023)** | | |
| --- | --- | --- |
| 1 | MH (“adolescence+” or “child+” or “infant+” or  TI or AB (infant disease* or childhood disease*) or  TI or AB adolescen* or teen* or  TI or AB (babies or baby or infant* or neonat* or neonat* or newborn* or new-born* or juvenil* or kid?) or school* or toddler? or underage? or under-age? or youth*)  TI or AB (boy? or child* or girl? or minors or minors* or  TI or AB (paediatric* or peadiatric* or pediatric*) or  TI or AB (perinat* or preschool* or puber* or pubescen*) | Population #1 |
| 2 | (MH "Hereditary Diseases+") or  TX (germline N2 mutation) or  TI or AB germline germline or  (MH "Neoplastic Syndromes, Hereditary+") or  TI or AB "familial cancer" or  TI or AB "cancer N2 predisposition" | Concept #1 |
| 3 | (MH "Team Nursing") or  (MH "Nurses+") or  (MH "Nursing Care Delivery Systems+") or  (MM "Advanced Practice Registered Nurses") or  (MH "Clinical Nurse Specialists") or  TI or AB "nurse or nurses or nursing" | Concept #2 |
| 4 | TI or AB “care coordination” or  TI or AB Surveilla or  (MH "Health Care Delivery, Integrated")  or  (MH "Disease Management+") or  (MH "Multidisciplinary Care Team+") or  (MH "Managed Care Programs+") or  TI or AB "Patient Care Planning" or  TI or AB "model? N2 care" or  (MH "Continuity of Patient Care") or  (MH "Transition to Adulthood") | Concept #3 |
| 5 | (MH "Health Services+") or  TX "genetic N2 service*" or  TX clinic or  TX hospital or  treatment N2 (centre OR center) | Context |
| 6 | 1 and 2 and (3 or 4) and 5 |  |
| 7 | limit 6 to yr="1991 - 2023" |  |
